# Comorbid Ankylosing Spondylitis and Systemic Lupus Erythematosus: A Therapeutic Challenge

**DOI:** 10.7759/cureus.57080

**Published:** 2024-03-27

**Authors:** Charles E DeBoisblanc, Victor E Nava, Suman Chauhan, Joyce Joseph

**Affiliations:** 1 Internal Medicine, George Washington University, Washington, DC, USA; 2 Pathology, George Washington University, Washington, DC, USA; 3 Pathology, Veterans Affairs Medical Center, Washington, DC, USA; 4 Rheumatology, Veterans Affairs Medical Center, Washington, DC, USA

**Keywords:** spondyloarthritis, disease-modifying antirheumatic drugs, rheumatology, systemic lupus erythematosus, ankylosing spondylitis

## Abstract

Ankylosing spondylitis (AS) and systemic lupus erythematosus (SLE) are common rheumatologic ailments that cause multiorgan system disease. The incidence of lupus and AS in the same patient is rare and has seldom been described in the literature. Comorbid lupus and AS provide interesting diagnostic and therapeutic challenges. Here, we present a case of comorbid lupus and AS, discuss the diagnostic challenges in diagnosing these conditions, and put forth possible therapeutic interventions that may benefit similar patients.

## Introduction

Ankylosing spondylitis (AS) and systemic lupus erythematosus (SLE) are complex conditions with multiorgan involvement, and both diseases are fairly common in rheumatologic practice. The estimated incidence of SLE and AS are 4.71-5.5/100,000 [1,2] and 5.4-7.3/100,000 [3,4] person-years, respectively. Though mild sacroiliitis can be seen in patients with lupus [5,6], a diagnosis of both lupus and AS in the same patient is uncommon and has been described only in a handful of case reports in the medical literature [7-13]. Comorbid SLE and AS provide interesting diagnostic and therapeutic challenges. In this case report, we present a patient with both of these diseases and discuss the challenges of concurrent management.

## Case presentation

History and physical assessment

A 50-year-old man with a past medical history of hypertension, osteoarthritis, chronic lower back pain, and posttraumatic stress disorder presented to a private rheumatology practice with polyarthralgia and joint effusions. He was preliminarily diagnosed with seronegative rheumatoid arthritis and was treated with methotrexate (MTX) and prednisone with significant symptomatic improvement. Etanercept was added to his regimen, with some improvement in his symptoms. Eight months before establishing care with our practice, he developed a scaly facial rash. Two months prior, he suffered an atraumatic rupture of his Achilles tendon. He had not been on fluoroquinolone antibiotics preceding the rupture. He transitioned care to our clinic for a second opinion. During the interview, the patient complained of continued joint pain accompanied by joint swelling as well as a recurrent facial rash.

More specifically, his joint pain involved bilateral wrists, left knee, bilateral ankles, and the left third proximal interphalangeal joint. Though these arthralgias were not daily, he had pain in at least one of these joints weekly. His joint pains improved with ibuprofen, of which he took three 800 mg doses daily and cyclobenzaprine nightly. If he missed a dose of ibuprofen or took his MTX a day or two late, he noticed his symptoms would increase in intensity. He experienced 20 minutes of associated morning stiffness that improved with movement. His chronic lower back pain had not changed in intensity for the past year. In addition, he reported that his third proximal interphalangeal joint would swell “like a sausage.”

**Figure 1:**
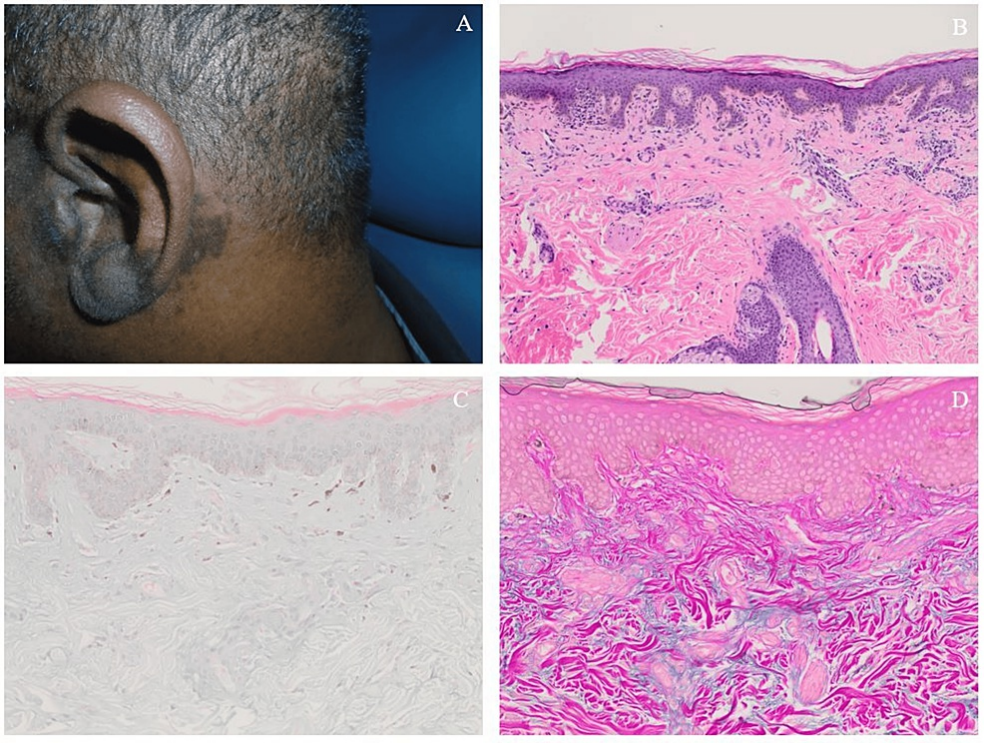
(A) Scaly, hyperpigmented macular rash in the region surrounding the patient’s auricle. (B) H&E stain of the lesion biopsy at 20x magnification, revealing perivascular inflammation. (C,D) Photomicrographs of the lesion biopsy, viewed at 40x magnification with Alcian blue (C) and colloidal iron (D) stains, demonstrating increased mucin deposition.

When describing the rash, the patient indicated the rash was recurrent, transient, and scaly, and it affected his ears and nose. When the rash manifested, it would last for one week and then begin to heal. The rash improved somewhat with moisturizer. On review of symptoms, he denied fevers, night sweats, dry eyes, dry mouth, mucosal ulcers, chest pain, cough, dyspnea, abdominal pain, myalgias, dysuria, and hematuria. He denied alcohol, tobacco, and illicit drug use, as well as a family history of autoimmune disease. He had served in the military, sustaining only minor injuries during training. His surgical history was notable only for the repair of his atraumatic Achilles tendon rupture in the year preceding the development of his current symptoms. On his initial visit, a physical examination revealed a normal cardiorespiratory system. His full joint exam was negative for synovitis. The dermatologic exam was notable for scaly plaques to the nasal apex, auricles, and external ear canals (Figure 1, Panel A).

Laboratory and imaging tests

**Figure 2:**
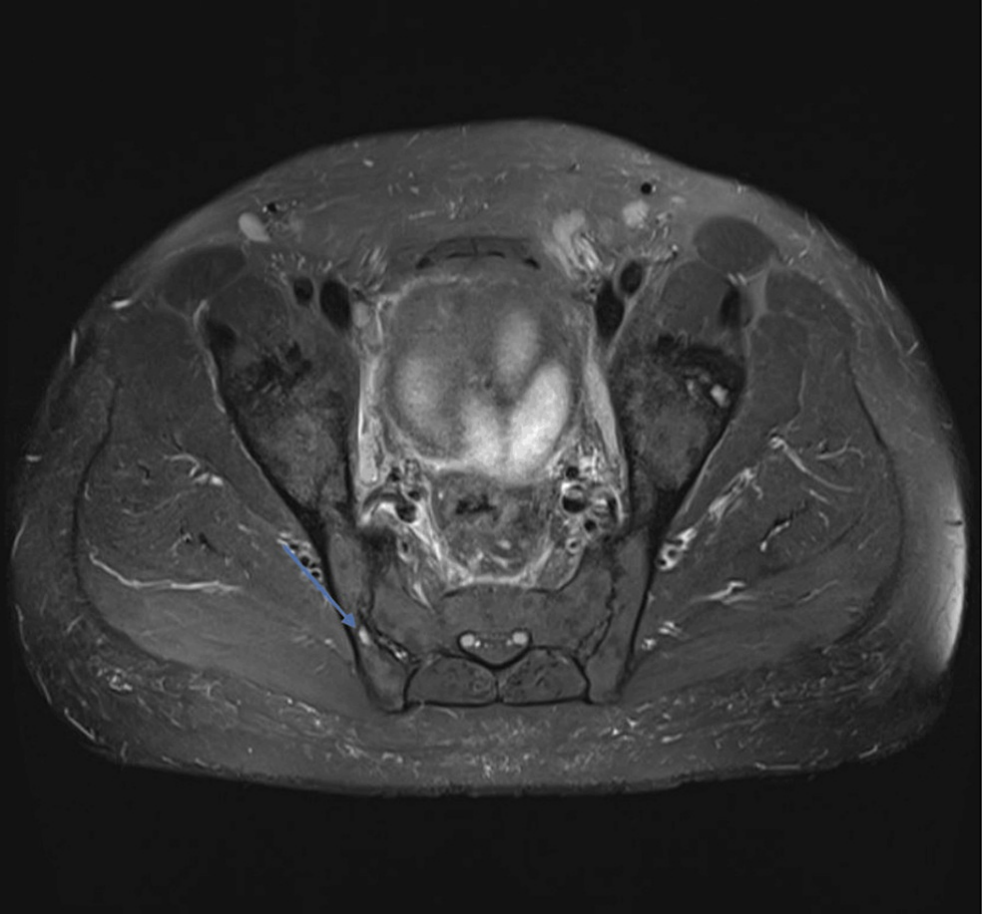
Sclerosis and joint space narrowing are present in the anterior aspect of the right cartilaginous portion of the right SI joint, along with a subcortical T2 hyperintense lesion (arrow) SI: Sacroiliac.

Initial laboratory tests in our clinic were notable for negative rheumatoid factor and anti-citrullinated peptide. His labs also revealed a urinalysis with urine protein of 90 mg/dL, a urine protein:creatinine ratio of 0.3 mg/g, hypocomplementemia (C3 68 mg/dL, C4 13 mg/dL), positive antinuclear antibody (1:1280, homogenous pattern), double-stranded DNA antibody levels > 1,000 units/mL (via multiplex flow immunoassay, Quest Diagnostics, Chantilly, VA), positive anti-histone antibodies, leukopenia (2.9 x 10^9^ cells/L, normal > 4.5 x 10^9^ cells/L), an elevated erythrocyte sedimentation rate (90 mm/hr, normal < 15 mm/hr), and a normal C-reactive protein (0.89 mg/dL, normal < 1 mg/dL). Human leukocyte antigen-B27 was negative. His HIV test was negative. A pelvic MRI revealed findings of partial fusion of the left sacroiliac (SI) joint, sclerosis, joint space narrowing of the anterior aspect of the cartilaginous portion of the right SI joint, and subcortical T2 hyperintensity in the right iliac bone (Figure 2).

Plain films of the hands, feet, and knees revealed changes consistent with degenerative disease but not with inflammatory arthropathy. Postauricular punch biopsy of his rash was significant for chronic perivascular inflammation, increased mucinosis, and few dermal melanophages (Figure 1, Panels B-D). The biopsy was then evaluated using immunohistochemistry for CD123, a plasmacytoid dendritic cell marker, which revealed increased perivascular plasmacytoid dendritic cell infiltration (data not shown). More recently, the patient also underwent a renal biopsy for proteinuria, with light microscopy findings consistent with class I lupus nephritis (International Society of Nephrology/Renal Pathology Society criteria, 2003) [14].

Differential diagnosis

The patient had an interesting constellation of symptoms that were not clearly attributable to a single disease. His arthralgias were predominantly symmetric and accompanied by morning stiffness in a pattern typical of rheumatoid arthritis. However, no evidence of chronic inflammatory arthritis was detected on x-rays. The skin biopsy findings from light microscopy were compatible with lupus dermatitis. This was supported by a review from an expert dermatopathologist and by further immunohistochemical staining for CD123. This stain showed perivascular infiltration of plasmacytoid dendritic cells, which has been reported in association with lupus dermatitis [15]. Combined with a strong-positive antinuclear antibody titer and markedly elevated anti-double-stranded DNA levels, leukopenia, hypocomplementemia, and joint symptoms without erosions, the patient met the American College of Rheumatology/European League Against Rheumatism criteria for SLE [16], which explained most of his symptoms. Drug-induced lupus secondary to etanercept exposure was also considered. Anti-tumor necrosis factor (anti-TNF) agents can rarely cause a lupus-like syndrome, and some of the patient’s described symptoms (serologic abnormalities, cytopenias, and arthralgias) have been demonstrated in few past observational studies [17,18]. In addition, the patient was found to be seropositive for anti-histone antibodies, which are classically associated with drug-induced lupus. However, his joint symptoms began before etanercept initiation and did not resolve with discontinuation. Also, unlike other medications associated with drug-induced lupus, anti-TNF agents are not strongly associated with anti-histone antibodies [17]. Furthermore, drug-induced lupus rarely involves the kidneys [19], and our patient was found to have class I lupus nephritis on renal biopsy. Therefore, based on collective findings, the most likely diagnosis is de novo lupus before starting etanercept. However, SI joint involvement was not in keeping with a diagnosis of lupus.

In addition to radiographic evidence of SI joint space narrowing and active inflammation on MRI, the patient had a history of peripheral synovitis, enthesitis (atraumatic rupture of Achilles tendon), and a good response to nonsteroidal inflammatory treatment. This was most consistent with AS. This diagnosis was supported by the 2009 Assessment of Spondyloarthritis International Society criteria for spondyloarthritis [20]. There was some concern for psoriatic arthritis or seborrheic dermatitis, given the scaly appearance of his rash, but the rash was not deemed to be consistent with either disease on review of the biopsy. In addition, his HIV test was negative. The lack of typical psoriatic rashes, no known history of inflammatory bowel disease, and no recent infections rendered psoriatic arthritis, inflammatory bowel disease-associated arthritis, and reactive arthritis less likely diagnoses. Therefore, it was deemed that AS was the most compatible diagnosis for his lower back pain despite his negative human leukocyte antigen B27.

Management

Lifestyle modifications, such as sun protection, were emphasized with the patient. The patient was discontinued from his MTX and etanercept since the diagnosis of rheumatoid arthritis was supplanted by SLE and AS. Initial lupus treatment included hydroxychloroquine 200 mg twice daily and prednisone 5 mg daily. The patient had two lupus flares, the first of which was characterized by pleurisy. The first flare was managed with colchicine 0.6 mg and prednisone started at 20 mg daily and tapered over weeks to his base dose of 5 mg. The colchicine was discontinued as it was not found to confer any benefit to the patient. After recovery from this first flare, secukinumab 300 mg each month was initiated for better AS control. He presented in the clinic four months later with polyarthralgia, SI joint tenderness, and bilateral knee effusions. Combined with elevated double-stranded DNA levels, C-reactive peptide, erythrocyte sedimentation rate, and hypocomplementemia, the patient was diagnosed with a lupus flare. Again, the patient was treated with a prednisone taper and started on azathioprine 150 mg daily, attaining symptomatic improvement in his lupus symptoms and lower back stiffness.

Despite overall improvement, some complications were observed during treatment initiation. Before starting secukinumab, he had a workup for his proteinuria, including a kidney biopsy. The biopsy was consistent with class I lupus nephritis. Also, an atraumatic rupture of the contralateral Achilles tendon occurred and was surgically managed successfully. Currently, close monitoring and treatment optimization are in progress (Table 1).

**Table 1:** Serial laboratory values are displayed with corresponding disease or management changes

Month	1	3	7	10	12	14	18	21
Erythrocyte sedimentation rate (mm/hour)	37	110	108	69	30	31	18	30
C-reactive protein (mg/dL)	0.89	1.95	9.24	8.24	1.24	0.85	0.1	0.8
Anti-double-stranded DNA (international units/dL)	1000	1000	1000	1000	1000	1000	1000	785.7
C3 (mg/dL)	68	68	71	69	62	57	81	87
C4 (mg/dL)	13	15	14	13	11	13	16	18
Urine protein (mg/dL)	90	50	300	50	70	50	30	50
Urine creatinine (mg/dL)	264.9	139	108	107.6	142.1	105.1	103.8	134.6
Urine protein:creatinine ratio from spot urine (mg/g)	0.3	0.4	2.8	0.5	0.5	0.5	0.3	0.4
Event	Systemic lupus erythematosus and ankylosing spondylitis are diagnosed.	The patient is admitted with lupus pleurisy and treated with prednisone taper.	The patient is admitted to the hospital for a urinary tract infection complicated by sepsis.	Lupus nephritis is diagnosed. The patient is started on azathioprine and treated for flare with a prednisone taper, with an inability to decrease the prednisone dose below 10 mg per day.	The increased dose of prednisone at 10 mg is maintained.	Azathioprine dosing is increased to 150 mg daily and secukinumab to 300 mg monthly.	The patient presents for routine follow-up.	The patient presents for routine follow-up. A slow prednisone taper of 1 mg/month is initiated.

## Discussion

In this case report, we present a patient who carries concomitant diagnoses of SLE and AS. AS disease activity is marginally affected by traditional disease-modifying antirheumatic drugs. Previous trials have shown that MTX and leflunomide are ineffective at addressing axial disease symptoms and progression [21,22]. Nonsteroidal anti-inflammatories remain the initial medication of choice for AS [23]. This may be problematic for patients with both AS and SLE, given the nephrotoxic potential of nonsteroidal anti-inflammatories and the frequency of renal involvement in lupus [24]. Biologics have become the mainstay of the management of AS refractory to nonsteroidal anti-inflammatory drugs. Anti-TNF agents, such as infliximab and adalimumab, have shown marked efficacy in reducing both disease activity and symptom severity in AS [23]. Interleukin-17a inhibitors, such as secukinumab, and interleukin-12/23 inhibitors, such as ustekinumab, have also demonstrated significant benefits in controlling SLE disease activity and symptoms [25,26]. However, none of the aforementioned biologics have proven efficacious in treating joint disease secondary to lupus. Management of lupus usually involves the antimalarial drug hydroxychloroquine as a first-line agent as well as traditional disease-modifying antirheumatic drugs as second-line agents [27,28]. Hydroxychloroquine is recommended in all patients with SLE but has not been demonstrated to be effective for AS [29].

Janus kinase (JAK) inhibitors are emerging as a possible therapeutic modality that may benefit patients with both AS and SLE. For example, baricitinib, a JAK 1/2 inhibitor, was found to be effective at suppressing SLE disease activity in phase II trials [30] but unfortunately has been inconsistent in phase III trials [31,32]. Recently, the JAK inhibitor tofacitinib was approved by the FDA for patients with ankylosing spondylitis [33], which, along with other JAK inhibitors, demonstrates the potential for treating refractory disease [34,35]. JAK inhibitors must be used cautiously for patients with comorbid AS and SLE due to the increased risk of therapy-related cancer and major adverse cardiac events in comparison to other agents [36].

## Conclusions

In conclusion, AS and SLE can rarely coexist in the same patient. When they do, this combination of conditions can impose some diagnostic challenges. This can render the selection of the patient's ideal treatment regimen challenging, but the provider should take into consideration drugs that may be able to treat both diseases concomitantly.
